# Systemic inflammation is associated with malaria and preterm birth in women living with HIV on antiretrovirals and co-trimoxazole

**DOI:** 10.1038/s41598-019-43191-w

**Published:** 2019-05-01

**Authors:** Chloe R. McDonald, Andrea M. Weckman, Andrea L. Conroy, Peter Olwoch, Paul Natureeba, Moses R. Kamya, Diane V. Havlir, Grant Dorsey, Kevin C. Kain

**Affiliations:** 1 SAR Laboratories, Sandra Rotman Centre for Global Health, University Health Network-Toronto General Hospital, Toronto, Ontario Canada; 2 Department of Laboratory Medicine and Pathobiology, University of Toronto, Toronto, Ontario Canada; 3 Department of Pediatrics, Indiana University, School of Medicine, Indianapolis, Indiana USA; 4 Makerere University-University of California, San Francisco Research Collaboration, Kampala, Uganda; 5 Department of Medicine, University of California San Francisco, San Francisco, California USA; 6 Tropical Disease Unit, Division of Infectious Diseases, Department of Medicine, University of Toronto, Toronto, Ontario Canada; 7 Toronto General Research Institute, Toronto General Hospital, Toronto, Ontario Canada

**Keywords:** Malaria, Acute inflammation, Reproductive biology, HIV infections

## Abstract

Women living with HIV (WLHIV) have an increased risk of malaria in pregnancy (MiP). It is unclear if MiP in WLHIV causes a systemic inflammatory response and increases the risk of adverse birth outcomes, especially for women receiving antiretroviral therapy (ART) and daily trimethoprim-sulfamethoxazole (TMP/SXT). We analyzed repeated plasma samples in a cohort of malaria-exposed Ugandan WLHIV receiving ART and daily TMP/SXT to examine changes in inflammatory markers across pregnancy and their association with birth outcomes. Concentrations of CHI3L1, CRP, IL-18BP, IL-6, sICAM-1, and sTNFR2 were quantified by ELISA in 1115 plasma samples collected over pregnancy from 326 women. MiP was associated with increased sTNFR2, sICAM-1 and IL-18BP concentrations across pregnancy. Women who delivered preterm had elevated concentrations of sTNFR2 and altered levels of IL-6 during pregnancy. Women with sTNFR2 concentrations in the highest quartile within 6 weeks of delivery had an increased relative risk of preterm birth. Our results indicate that despite daily TMP/SXT, MiP in WLHIV induced a systemic inflammatory response that was associated with an increased risk of preterm birth. These findings highlight the need for additional strategies to protect WLHIV from malaria infection in pregnancy to promote healthy outcomes for mother and child.

## Introduction

Two thirds of all people living with HIV reside in sub-Saharan Africa. In sub-Saharan Africa, HIV and malaria co-infection poses an important health risk for pregnant women. Young women (age 15–24 years) make up 11% of the adult population and 20% of new HIV infections^1^. Cultural, social, and economic inequality has resulted in women of reproductive age representing almost half of all adults living with HIV worldwide^2^. Increased use of WHO-recommended multi-drug antiretroviral therapy (ART) during pregnancy has improved health outcomes in women living with HIV (WLHIV). Rates of pregnancy in WLHIV are comparable to the general population^3–5^; however, risk of adverse birth outcomes, including preterm birth (PTB), small-for-gestational age, low birth weight, and stillbirth, remain higher in WLHIV^6^, irrespective of ART^2^.

The risk of a poor birth outcome is increased for pregnant women living in malaria-endemic regions. An estimated 85 million women a year are at risk of *Plasmodium falciparum* malaria in pregnancy (MiP)^7^. Pregnant women are more likely to be infected with malaria and to experience more severe disease^8,9^. Women with MiP are at higher risk for adverse birth outcomes^10^. Recommended care for pregnant women living in malaria-endemic regions includes the use of insecticide-treated bed nets (ITNs), intermittent preventive treatment of malaria in pregnancy (IPTp, beginning in the second trimester), and clinical management of MiP and anemia. However, low rates of ITN coverage, increasing resistance to IPTp drugs, and limited access to antenatal care are important barriers to impact, and MiP remains a global health priority.

For many sub-Saharan African women of reproductive age, co-infection with HIV and MiP further increases the risk of poor birth outcomes. WLHIV have an increased incidence and density of peripheral and placental malaria infection, and experience more complications including maternal anemia and maternal death^11^. Co-infection with malaria and HIV is associated with an increased risk of PTB and low birth weight compared to the risk from either infection alone^11^. An estimated 15 million children are born preterm every year, resulting in over one million deaths in children under the age of five^12^. The majority of all PTBs - over 60% - occur in sub-Saharan Africa and South Asia. This high prevalence of PTB coincides with high disease burden in pregnancy, including the risk of HIV and malaria infection. An enhanced understanding of the systemic response to co-infection during pregnancy may inform new interventions to reduce adverse birth outcomes, including PTB.

Systemic inflammation is a leading risk factor for spontaneous PTB^13^. Here we examined changes in inflammatory markers over the course of pregnancy in a cohort of WLHIV in Uganda who were receiving ART as well as daily prophylactic treatment with trimethoprim-sulfamethoxazole (TMP/SXT). Due to its burden of disease in sub-Saharan Africa and the limited data on PTB in women at risk for MiP, PTB was our primary outcome of interest. We examined systemic inflammation across pregnancy in association with MiP and the risk of PTB.

## Results

### Study cohort

All women were ART naïve at enrollment before randomization to receive Lopinavir/ritonavir (LPV/r)- or Efavirenz (EFV)-based ART. No differences in demographics were observed between treatment arms (Supplementary Table S1); therefore, the following data for the combined cohort are presented. Most women were multigravid (52.4% had >four previous pregnancies), with a median age of 30 years and 23.6 weeks of gestation at enrollment (Table 1). At enrollment, median CD4 count was 369 (interquartile range [IQR] 271–504) cells/mm^3^, and median HIV RNA viral load was 15.5 (IQR 2.4–59.4) log_10_ copies/mL (Table 1). In this cohort, 16.9% of deliveries were preterm (the national estimate of PTB in Uganda is 14%). MiP was not associated with an increased frequency of PTB. No women in the study cohort were diagnosed with hypertension, preeclampsia, or eclampsia for the duration of the study.

**Table 1:** Descriptive characteristics of the study population.

Baseline Characteristics n(%) or median[IQR]		
Age (years)		30 [26,33]
BMI (kg/m^2^)		21.4 [19.9, 23.0]
Socioeconomic status (tertile)	1	111 (35.9)
	2	135 (43.7)
	3	63 (20.4)
Gestational age enrolment (weeks)		23.57 [19.6, 27.9]
Previous pregnancies	≤2	55 (16.9)
	3–4	100 (30.7)
	5–6	108 (33.1)
	≥7	63 (19.3)
Hemoglobin level (g/dL)		11 [10.2,11.8]
White blood cell count (cells/mm^3^)		5050 [4200, 6200]
Platelet count (×10^9^/L)		210 [173,252]
CD4+ T-cell count (cells/mm^3^)		369 [271,504]
HIV RNA load (log_10_copies/mL)		15.5 [2.4,59.4]
**Perinatal Characteristics**		
Gestational age delivery (weeks)		38 [37, 40]
Birth weight (g)		2890 [2670,3230]
Preterm birth		55 (16.9)
Small-for-gestational age		81 (25.6)
Stillbirth		9 (2.8)
**Malaria Status**		
Antenatal peripheral blood smear^a^		25 (7.9)
Placental PCR		24 (8.9)
Placental histology		96 (33)
Placental blood smear		10 (3.6)
Placental rapid diagnostic test		12 (4.4)
Any evidence of malaria in pregnancy^b^		119 (40)

^a^Number of women with at least 1 positive antenatal blood smear. ^b^As evidenced by positive antenatal blood smear; placental histology, PCR, blood smear or rapid diagnostic test. n (%) expressed as percent of women with existing data for respective variable.

Abbreviations: IQR, interquartile range; BMI, body mass index; PCR, polymerase chain reaction.

### Immunological status at enrolment was associated with risk of malaria infection

40% of women had evidence of malaria infection during pregnancy as determined by antenatal peripheral blood smear, and/or placental histology, blood smear, rapid diagnostic test and/or PCR (Table 1). The majority of malaria diagnoses were made by placental histopathology (33%). We examined markers of immunological status at enrolment (viral load, CD4, white blood cell and neutrophil count) and the relative risk (RR) of MiP. Women with higher white blood cell (RR = 0.51; 95% confidence interval (CI) 0.26, 0.97; p = 0.04) and neutrophil (RR = 0.60; 95% CI 0.40, 0.89; p = 0.01) counts had a reduced relative risk of MiP (Table 2). There was no association between white blood cell or neutrophil count at enrolment and PTB.

**Table 2:** Relative risk of malaria infection in pregnancy according to enrollment immunological status.

Immunological Status Marker	Univariate RR (95% CI)	Multivariate RR (95% CI)
Viral Load	1.05 (0.96, 1.14)	1.06 (0.97, 1.15)
CD4 Count	1.24 (0.89, 1.77)	1.21 (0.86, 1.73)
White Blood Cell Count	0.57 (0.30, 1.08)	0.51 (0.26, 0.97)^a^
Neutrophil Count	0.63 (0.43, 0.95)^a^	0.60 (0.40, 0.89)^a^

Relative risks and 95% CI were estimated using log-linked binomial models. Log-linked poisson models were used when models did not converge. Multivariate models adjusted for gravidity and maternal age. ^a^p < 0.05.

Abbreviations: RR, relative risk; CI, confidence intervals.

### Inflammatory markers by ART treatment arm

**Figure 1:**
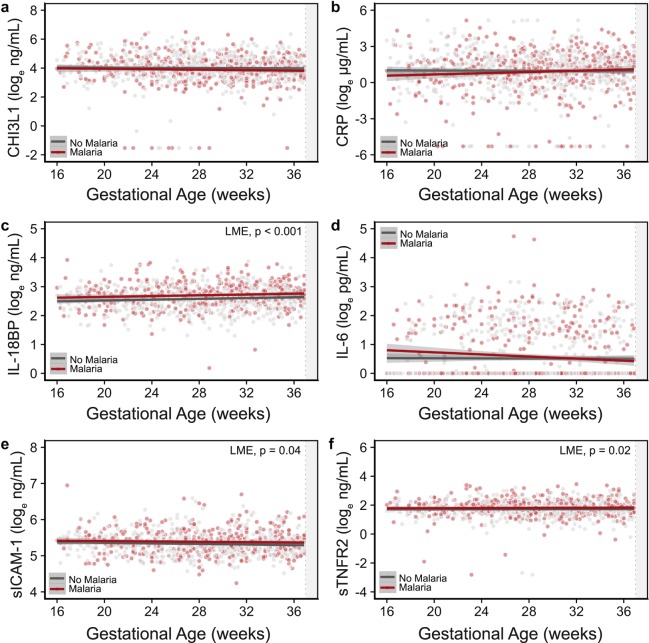
Longitudinal changes in inflammatory markers across pregnancy by malaria status. Logged (natural log) plasma concentrations of (**a**) CHI3L1 (ng/mL), (**b**) CRP (μg/mL), (**c**) IL-18BP (ng/mL), (**d**) IL-6 (pg/mL), (**e**) sICAM-1 (ng/mL), and (**f**) sTNFR2 (ng/mL) by gestational age of sample collection. Abbreviations: CHI3L1, chitinase-3-like 1; CRP, C-reactive protein; IL-6, interleukin-6; IL-18BP, interleukin 18 binding protein; sICAM-1, soluble intercellular adhesion molecule-1; sTNFR2, soluble tumor necrosis factor receptor-2.

A median number of three (IQR 2–4) samples were tested per participant (Supplementary Table S2). No longitudinal differences in concentrations of chitinase-3-like 1 (CHI3L1), interleukin-18 binding protein (IL-18BP), interleukin-6 (IL-6), soluble intercellular adhesion molecule-1 (sICAM-1), or soluble tumor necrosis factor receptor 2 (sTNFR2) were reported by treatment arm (Supplementary Table S3, Fig. 1). Women receiving EFV-based ART had higher levels of C-reactive protein (CRP) across pregnancy, in comparison with women receiving LPV/r-based ART (Supplementary Table S3, Fig. 1). All subsequent linear mixed effects (LME)-based analysis adjusted for treatment group to account for the parent trial design. The parent study did not report differences in the risk of MiP or in birth outcome (spontaneous abortion or stillbirth, PTB, low birth weight or composite adverse birth outcome) between treatment arms^14^.

### Changes in systemic inflammation were associated with malaria infection and the risk of preterm birth

**Figure 2:**
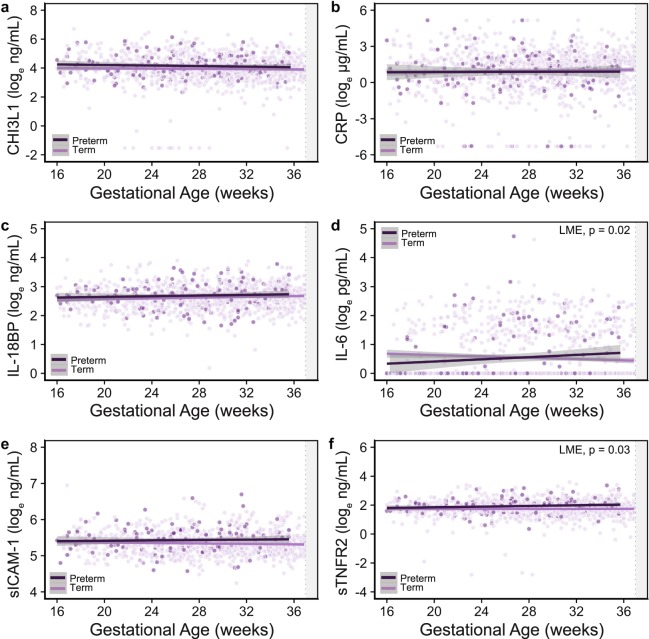
Longitudinal changes in inflammatory markers across pregnancy by preterm birth status. Logged (natural log) plasma concentrations of (**a**) CHI3L1 (ng/mL), (**b**) CRP (μg/mL), (**c**) IL-18BP (ng/mL), (**d**) IL-6 (pg/mL), (**e**) sICAM-1 (ng/mL), and (**f**) sTNFR2 (ng/mL) by gestational age of sample collection. Abbreviations: CHI3L1, chitinase-3-like 1; CRP, C-reactive protein; IL-6, interleukin-6; IL-18BP, interleukin 18 binding protein; sICAM-1, soluble intercellular adhesion molecule-1; sTNFR2, soluble tumor necrosis factor receptor-2.

To examine the impact of MiP in WLHIV on systemic inflammation we quantified longitudinal changes in inflammatory proteins by gestational age and malaria status (Fig. 1). MiP was associated with elevated sTNFR2 (p = 0.02), sICAM-1 (p = 0.04) and IL-18BP (p < 0.001) across pregnancy (Fig. 1, Supplementary Table S4). No significant differences were observed in the concentrations of CHI3L1, CRP or IL-6 by malaria infection status (Fig. 1). Based on the role of infection-induced inflammation in PTB we examined longitudinal changes in inflammatory proteins in preterm and term deliveries (Fig. 2). Women who went on to deliver preterm had elevated concentrations of sTNFR2 (p = 0.03) and altered levels of IL-6 (p = 0.02) over the course of pregnancy (Fig. 2, Supplementary Table S5). Levels of sTNFR2 were elevated across pregnancy, while levels of IL-6 showed an interaction with gestational age, rising across pregnancy in preterm deliveries and falling across pregnancy in term deliveries (Fig. 2). There were no associations between inflammatory markers across pregnancy and small-for-gestational age or stillbirth outcomes. Due to its distribution, IL-6 could not be analyzed by quartile. When we examined analyte concentrations in the sample collected closest to delivery (within six weeks of delivery), we observed an increased relative risk of PTB in women with sICAM-1 (RR = 2.47; 95% CI 1.01, 6.59; p = 0.055) and sTNFR2 (RR = 3.30; 95% CI 1.27, 10.00; p = 0.02) in the highest quartile (Table 3).

**Table 3:** Multivariate relative risk of preterm birth according to biomarker levels by quartile in sample collected closest to delivery.

	Quartile 1	Quartile 2	Quartile 3	Quartile 4
CHI3L1	1.00 (Reference)	1.21 (0.48, 3.12)	1.55 (0.62, 4.05)	1.49 (0.65, 3.59)
CRP	1.00 (Reference)	0.52 (0.22, 1.18)	0.69 (0.30, 1.52)	0.58 (0.22, 1.41)
IL-18BP	1.00 (Reference)	1.40 (0.59, 3.50)	1.18 (0.46, 3.10)	1.94 (0.65, 6.38)
IL-6^a^	1.00 (Reference)	—	—	—
sICAM-1	1.00 (Reference)	1.22 (0.45, 3.44)	1.49 (0.56, 4.01)	2.47 (1.01, 6.59)^b^
sTNFR2	1.00 (Reference)	1.26 (0.44, 3.72)	1.99 (0.83, 5.02)	3.30 (1.27, 10.00)^c^

Relative risks and 95% confidence intervals were estimated using log-poisson models. Multivariate models adjusted for gravidity, sample gestational age, maternal age, BMI at enrollment. ^a^Quartiles could not be generated based on the distribution of IL-6 concentration. ^b^p = 0.05, ^c^p = 0.02.

Abbreviations: CHI3L1, chitinase-3-like 1; CRP, C-reactive protein; IL-6, interleukin 6; IL-18BP, interleukin 18 binding protein; sICAM-1, soluble intercellular adhesion molecule-1; sTNFR2, soluble tumor necrosis factor receptor 2.

## Discussion

In this study we assessed the longitudinal concentrations of inflammatory mediators implicated in the pathobiology of adverse birth outcomes over the course of pregnancy in Ugandan WLHIV. We observed a systemic pro-inflammatory response associated with MiP, as well as an association between systemic inflammatory response and an increased risk of PTB in WLHIV receiving ART and daily chemoprophylaxis with TMP/SXT. Women who delivered preterm had higher circulating levels of sTNFR2 and altered kinetics of IL-6 across pregnancy. Women with concentrations of sICAM-1 and sTNFR2 in the highest quartile in samples collected less than six weeks prior to delivery had an increased relative risk of PTB.

MiP results in the accumulation of mononuclear cells and a localized inflammatory response in the placenta, which may extend to a systemic inflammatory response^15,16^. Studies assessing malaria during pregnancy have linked systemic inflammation to adverse birth outcomes, including low birth weight and PTB in HIV-negative cohorts^17^. Previous studies have linked MiP to increases in TNF in placenta plasma samples from HIV-seronegative women^16,18^ and to the *in vitro* production of IL-18 and CXCL-10/IP-10 in placental intervillous blood mononuclear cells from MiP-positive WLHIV^19^. However few studies have examined the impact of malaria and HIV co-infection on *in vivo* patterns of systemic inflammation over pregnancy and its association with birth outcomes.

Here we report increased circulating levels of sTNFR2, sICAM-1, and IL-18BP in WLHIV with MiP. We focused on sTNFR2 and IL-18BP concentrations, as these proteins have been shown to be more informative indicators of systemic inflammation than TNF or IL-18^20^. The activity of TNF is regulated by the TNF I and II receptors, the extracellular domains of which are cleaved from the cell surface to form soluble TNFR I (sTNFR1) and soluble TNFR II (sTNFR2). Concentrations of the soluble receptors are considered markers of TNF-mediated pro-inflammatory activity^21^. IL-18BP is an antagonist of pro-inflammatory IL-18. Levels of IL-18BP increase in response to elevated levels of circulating IL-18. The soluble adhesion molecule sICAM-1 is cleaved in response to inflammation and endothelial activation. Elevated circulating concentrations of sTNFR2, sICAM-1, and IL-18BP have been associated with various infections, as well as disease severity^21–23^. The elevated concentrations of sTNFR2, sICAM-1, and IL-18BP we observed in association with MiP in WLHIV, indicate a malaria-induced systemic inflammatory response. These findings are consistent with studies of MiP in HIV-uninfected women^17,24^.

Infections and the resulting inflammation are well-established risk factors for spontaneous PTB, although the precise pathobiology leading from inflammation to PTB is not fully understood^13,25^. Several lines of evidence link altered pro-inflammatory cytokine levels to the initiation of PTB, including the induction of myometrial contractions, preterm premature rupture of membranes (PPROM), and cervical maturation^13,25^. Here we observed altered kinetics of circulating IL-6 and sTNFR2 across pregnancy in women who subsequently delivered preterm. Pro-inflammatory factors including IL-6 are involved in regulating the intrauterine inflammatory response that contributes to fetal inflammatory response syndrome, membrane rupture, and PTB^25,26^. However, many studies examining the role of IL-6 in PTB have measured IL-6 only later in pregnancy (e.g., using amniotic fluid samples)^25^. Our data provide some of the first evidence for a maternal systemic IL-6 response in association with PTB in WLHIV. In our cohort, the median concentration of IL-6 early in pregnancy was initially lower in women who went on to deliver preterm, but then exceeded term pregnancy concentrations after approximately 26 weeks of gestation. Since women in spontaneous term labour have elevated circulating IL-6^27–29^, our data are consistent with a hypothesis that systemic inflammation can trigger premature initiation of physiological mechanisms leading to labour and delivery. Considerable evidence also links TNF to the pathology of PTB, including increased TNF concentrations in women in preterm labour and women with PPROM, as well as mechanistic roles for TNF in mediating membrane rupture, cervical ripening and infection-induced PTB^25^. In this cohort, MiP in WLHIV was associated with increased circulating levels of sTNFR2, IL-18BP, and sICAM-1 longitudinally. These findings are supported by similar observations in HIV-negative women, including an association between spontaneous PTB and elevated plasma sTNFR2 and sICAM-1 concentrations^20^. Although elevated IL-18BP has previously been associated with PTB^20^, it was not observed in this population. These data suggest that MiP-induced elevations in pro-inflammatory markers such as sTNFR2, IL-18BP, and sICAM-1, could disrupt the closely regulated balance of pro-and anti-inflammatory proteins required to maintain ongoing pregnancy, and thereby initiate pathways leading to PTB.

Notably, our results indicate that despite receiving ART and daily chemoprophylaxis with TMP/SXT, WLHIV had evidence of MiP together with systemic inflammation (sTNFR2, sICAM-1, and IL-18BP) across pregnancy and an increased risk of PTB. These data suggest that current WHO-recommended policies to prevent MiP may not provide optimal protection for WLHIV. While Tororo, Uganda is considered a high-transmission region for malaria, the prevalence of malaria in this study cohort was relatively low; however, women are still showing MIP-induced changes in inflammatory profiles. WHO recommends daily co-trimoxazole chemoprophylaxis during pregnancy for WLHIV to prevent malaria and opportunistic infections^30^. The addition of an effective antimalarial to daily TMP/SXT may improve protection against MiP^31–33^; however, the appropriate antimalarial and dosing regimen for pregnant WLHIV remains controversial. It is possible that women in this cohort had malaria infection prior to enrollment. However 64% reported using TMP/SXT prophylaxis prior to enrollment and the majority of participants were receiving prophylactic treatment throughout pregnancy^14^. Collectively the available evidence is insufficient and additional studies are required to determine a drug-based strategy to adequately prevent MiP in WLHIV and promote healthy birth outcomes for mother and child.

To our knowledge no previous study has reported longitudinal inflammatory profiles across pregnancy in a cohort of WLHIV at risk of MiP. Longitudinal sample collection allowed for the examination of changes in systemic inflammation in association with gestational age and malaria-infection status across pregnancy. This study had several strengths including the prospective design of the parent study, longitudinal collection of plasma samples, and the ability to control for multiple covariates, however there are also limitations. This is a retrospective analysis of a cohort of women enrolled in a randomized controlled trial. In the absence of an HIV-negative control group, it is not possible to examine the influence of HIV status on our observed systemic inflammatory response. We did not observe a direct association between MiP and PTB which may reflect the timing of malaria infection and diagnosis in pregnancy. In this study the majority of malaria diagnoses were made at delivery through histopathological examination of the placenta. In this study antenatal diagnosis was confirmed by blood smear and not PCR. Since blood smears are less sensitive than PCR, it is possible that they did not detect early malaria infections that could have triggered the persistent systemic inflammatory response associated with PTB that we observed. Given the small number of women with antenatal smear-positive malaria (n = 25) it was not possible to link birth outcome to the timing of malaria infection. Grouping all malaria infections may mask the effect that timing of infection has on the association between MiP and PTB. Further, as evidenced by the observation that changes in IL-6 were associated with PTB but not MiP, other factors for which we did not have data (i.e. intrauterine infection) could also have contributed to the association between systemic inflammation and PTB. Additional studies are required to examine longitudinal changes in inflammatory response in both HIV seropositive and seronegative women at risk of MiP. Further research is also required to establish how the timing of malaria infection during pregnancy impacts persistent systemic inflammatory responses and the risk of PTB^34^.

Collectively our results suggest that MiP in WLHIV initiates inflammatory pathways that can increase the risk of PTB. These findings are consistent with mechanistic studies linking inflammation to PTB and add to a growing body of evidence that malaria-induced systemic inflammation is associated with poor birth outcomes, including PTB. These findings indicate that current treatment strategies for WLHIV may be inadequate to prevent MiP and the resulting systemic inflammatory responses and associated adverse birth outcomes.

## Methods

### Study population and clinical procedures

This was a secondary analysis of women enrolled in an open-label, single-site, randomized control trial of LPV/r-based versus EFV-based ART conducted in Tororo, Uganda from December 2009 to March 2013^14^. HIV positive, ART-naïve women who were ≥16 years of age and pregnant (12–28 weeks of gestation by last menstrual period with ultrasound confirmation) were invited to participate. Women were eligible for enrollment at any CD4 cell count. Women who had received ART within the last 24 months were ineligible. Participants were randomized (1:1), following stratification by gravidity and gestational age at enrollment to receive LPV/r- or EFV-based ART. All participants received daily TMP/SXT (160 mg/800 mg). Blood collection took place at enrollment and all antenatal visits. MiP was diagnosed by blood smear at antenatal care visits, and at delivery by thick blood smear, PCR, malaria rapid diagnostic test, and by placental histology. The procedures for preparation and examination of placental specimens have been reported in detail^14^. All placental specimens were collected within 30 minutes of delivery. At this time a thick blood smear was collected, a rapid diagnostic test (Paracheck-Pf, Orchid, Goa, India) was performed, and a dried blood spot was collected for PCR. Placental histology was performed on a 2 × 2 full-thickness biopsy embedded in paraffin wax and sectioned into slides stained with hematoxylin-eosin (H&E) and Giemsa. A trained investigator performed all histopathological analysis, which included an examination of malaria parasites, hemozoin pigment in intervillous fibrin, and macrophages in accordance with standardized criteria^14^. A composite measure of MiP included any evidence of malaria infection in pregnancy by antenatal peripheral blood smear and/or at delivery identified by placental histology, PCR, blood smear and/or rapid diagnostic test. Standardized assessments were completed at delivery including gestational age and birth weight (using an electronic scale). PTB is defined as delivery prior to 37 completed weeks of gestation, as confirmed by ultrasound dating. Women were eligible for inclusion in the present study if they had a singleton pregnancy with known birth outcome, and samples collected within 6 pre-specified gestational age bins: 16– < 20, 20– < 24, 24– < 28, 28– < 32, 32– < 36, 36– < 37 weeks of gestation.

All women received standard antenatal care according to the Ugandan Ministry of Health Guidelines (Uganda Ministry of Health Clinical Guidelines, http://www.health.go.ug/docs/ucg_2010.pf). The study received ethical approval from Makerere University School of Medicine (Kampala, Uganda), the Uganda National Council for Science and Technology (Kampala, Uganda), the National Drug Authority (Kampala, Uganda), the University of California-San Francisco (San Francisco, California), and the University Health Network (Toronto, Canada). The trial was registered at ClinicalTrials.gov (identifier:NCT00993031). Signed informed consent was obtained from each participant enrolled in the clinical trial to allow for blood collection and subsequent analysis of plasma samples for inflammatory proteins.

### Protein quantification assays

The current study included all available plasma samples from women who met our inclusion criteria (n = 1115 plasma samples, n = 326 women). Maternal peripheral plasma samples were collected in EDTA and stored at −80 °C unthawed until testing. Plasma samples were processed using enzyme-linked immunosorbent assays (Duosets, R&D Systems, Minneapolis, MN) listed with dilution factors: CHI3L1 (1:1000), CRP (1:8000), IL-18BP (1:20), IL-6 (1:20), sICAM-1 (1:500), and sTNFR2 (1:200). The markers were selected based on previous studies reporting an association with MiP and PTB^20,24,35^. Processing and analysis of samples was performed according to the manufacturer’s instructions and blinded to treatment group and outcome.

### Statistical analysis

Statistical analysis was performed using STATA v12 (StataCorp, TX) and R version 3.5.1 (R Foundation for Statistical Computing, Vienna, Austria) software^36^. Descriptive data were summarized using median [IQR] or n (%). Baseline characteristics were compared between trial arms using the Mann-Whitney U test, Pearson Chi-square test, or Fisher’s exact test where appropriate. LME modeling (R package “lme4”, LME models using Eigen and S4; R package version 1.1–21)^37^ was used to examine longitudinal changes in log_e_-transformed protein concentrations across gestation by trial arm, malaria status, and PTB outcome. Log_e_–transformation was used as the concentrations of biomarkers showed deviations from normality (Shapiro-Wilks p < 0.05). The model included a random-intercept for each participant and a by-participant random slope for the effect of gestational age. We shifted the gestational age covariate to provide a meaningful intercept by subtracting the lowest gestational age from all gestational age values. All models included an interaction term between gestational age and treatment arm. Models adjusted for gestational age at blood sample collection, maternal age, body mass index (BMI: kg/m^2^), gravidity, and the interaction between treatment arm and gestational age. Visual inspection of residual plots did not reveal deviations from normality. Log-linked binomial regression models were used to estimate RR (with 95% CI) of malaria by immunological status (viral load, CD4, white blood cell, and neutrophil count), and PTB across quartiles of protein concentrations, with the lowest quartile as the reference category. In cases where the model did not converge, log-linked poisson regression models were used. RR of PTB by protein concentration in quartiles were estimated using the blood sample collected closest to the delivery date (up to a maximum of six weeks prior to delivery). All p-values were two-sided and values of <0.05 were considered statistically significant.

## Supplementary information

Supplementary Information

## Data Availability

Inflammatory marker data analyzed for this study are available from the corresponding author by reasonable request. Statistical output of linear mixed effects modeling is included in the Supplementary Information.
