# Supplementary material for: Systemic inflammation is associated with malaria and preterm birth in women living with HIV on antiretrovirals and co-trimoxazole
Source: Sci Rep. 2019 May 1;9:6758. doi: 10.1038/s41598-019-43191-w (PMC6494863; doi:10.1038/s41598-019-43191-w)
Supplement: Supplementary file 1 — Supplementary Information [file 41598_2019_43191_MOESM1_ESM.pdf]

SUPPLEMENTARY INFORMATION

Systemic inflammation is associated with malaria and preterm birth in women living with HIV on antiretrovirals and co-trimoxazole

Chloe R. Mcdonald, Andrea M. Weckman, Andrea L. Conroy, Peter Olwoch, Paul Natureeba, Moses Kamya, Diane V. Havlir, Grant Dorsey, and Kevin C. Kain

Supplementary Table S1: Descriptive characteristics of the study population by trial arm

		Efavirenz-Based ART	Lopinavir/ritonavir-Based ART	
Number of Participants		160	166	
**Baseline Characteristics**		**n(%) or median[IQR]**		**p-value**
Age (years)		30[26,33]	29[25,34]	0.62
BMI (kg/m^2)		21.2[19.6,22.9]	21.6[20.2,23.2]	0.07
Socioeconomic status (tertile)	1	54(48.7)	57(51.4)	0.61
	2	68(50.4)	67(49.6)	
	3	27(42.9)	36(57.1)	
Previous pregnancies	≤2	28(50.9)	27(49.1)	0.28
	3-4	46(46)	54(54)	
	5-6	59(54.6)	49(45.4)	
	≥7	27(42.9)	36(57.1)	
Hemoglobin level (g/dL)		11[10.2,11.8]	11.1[10.2,11.7]	0.86
White blood cell count (cells/mm^3)		4900[4100,6050]	5200[4300,6400]	0.13
Platelet count (x10^9/L)		218[178,255]	201[167,239]	0.08
CD4+T-cell count (cells/mm^3)		373[269.8,495.2]	368[281,505]	0.58
HIV RNA load (log_{10}copies/mL)		19.3[2.8,67.6]	12.5 [2.2,56.4]	0.47
**Perinatal Characteristics**				
Gestational age delivery (weeks)		39[37,40]	38[37,39]	0.06
Birth weight (kg)		2910[2680,3240]	2880[2650,3210]	0.50
Preterm birth		24(15.0)	31(18.7)	0.46
Small-for-gestational age		37(23.9)	44(27.1)	0.52
Stillbirth		4(2.5)	5(3.0)	1.00
**Malaria Status**				
Antenatal peripheral blood smear		15(9.7)	10(6.2)	0.30
Placental PCR		14(10.4)	10(7.5)	0.52
Placental histology		40(27.4)	56(38.6)	0.05
Placental blood smear		6(4.4)	4(2.9)	0.54
Placental rapid diagnostic test		7(5.2)	5(3.6)	0.57
Any evidence of malaria in pregnancy[a]		52(34.9)	67(44.4)	0.12

[a]As evidenced by positive antenatal blood smear and/or placental histology, PCR, blood smear or rapid diagnostic test.
Abbreviations: ART, antiretroviral therapy; BMI, body mass index; IQR, interquartile range; PCR, polymerase chain reaction.

Supplementary Table S2: Flow chart of participants included in the study analysis.

Gestational Age Category of Plasma Sample	NNRTI, n = 160 Efavirenz-based ART	PI, n = 166 Lopinavir/ritonavir-based ART	Total Cohort, n = 326 participants
	Number of Samples Processed		Total Samples
16 to <20 weeks	45	48	93
20 to <24 weeks	77	80	157
24 to <28 weeks	115	128	243
28 to <32 weeks	140	145	285
32 to <36 weeks	136	145	281
36 to <37 weeks	24	32	56
Total	537	578	1115

Abbreviations: ART, antiretroviral therapy; NNRTI, non-nucleoside reverse-transcriptase inhibitor; PI, protease inhibitor.

Supplementary Table S3: Linear mixed effects modeling of longitudinal changes in inflammatory proteins by parent trial treatment group.

	CHI3L1		CRP		IL-18BP		IL-6		sICAM-1		sTNFR2	
**Fixed Effects[a]**	**Estimate (SE)**	**T-Value**	**Estimate (SE)**	**T-Value**	**Estimate (SE)**	**T-Value**	**Estimate (SE)**	**T-Value**	**Estimate (SE)**	**T-Value**	**Estimate (SE)**	**T-Value**
(Intercept)	4.09 (0.08)	48.65	0.90 (0.13)	6.74	2.57 (0.03)	74.99	0.63 (0.07)	9.58	5.43 (0.03)	213.59	1.81 (0.04)	40.99
Gestational Age (Shifted)	-0.008 (0.005)	-1.74	0.015 (0.009)	1.62	0.004 (0.002)	1.59	-0.006 (0.005)	-1.16	-0.007 (0.0018)	-3.73	-0.004 (0.003)	-1.25
Gestational age; treatment arm interaction	-0.0001 (0.004)	-0.04	-0.018 (0.009)	-2.06	0.003 (0.003)	1.08	-0.003 (0.004)	-0.66	0.002 (0.002)	1.10	0.004 (0.003)	1.36
**Random Effects**	**Variance (SD)**	**----**	**Variance (SD)**	**----**	**Variance (SD)**	**----**	**Variance (SD)**	**----**	**Variance (SD)**	**----**	**Variance (SD)**	**----**
Participant (Intercept)	1.35 (1.16)	----	1.92 (1.39)	----	0.15 (0.39)	----	0.014 (0.12)	----	0.069 (0.26)	----	0.176 (0.42)	----
Gestational Age (Shifted)	0.0008 (0.03)	----	0.0006 (0.02)	----	0.0002 (0.014)	----	0.0003 (0.016)	----	0.00001 (0.003)	----	0.0001 (0.011)	----
Residual	0.33 (0.58)	----	1.59 (1.26)	----	0.068 (0.26)	----	0.725 (0.85)	----	0.05 (0.22)	----	0.187 (0.43)	----
Observations (n)	1115	----	1115	----	995	----	1115	----	995	----	1115	----
Participants (n)	326	----	326	----	295	----	326	----	295	----	326	----
LR test against null model	$X^2(1) = 0.0013$, p = 0.97		$X^2(1) = 4.18$, **p = 0.04**		$X^2(1) = 1.17$, p = 0.28		$X^2(1) = 0.43$, p = 0.51		$X^2(1) = 1.19$, p = 0.27		$X^2(1) = 1.86$, p = 0.17	

Linear mixed effects modeling to evaluate changes in inflammatory proteins across pregnancy by treatment group (Lopinavir/ritonavir (LPV/r)-based versus Efavirenz (EFV)-based antiretroviral therapy. [a]Models adjusted for gestational age at sample collection and the interaction between gestational age and treatment arm. Abbreviations: CHI3L1, chitinase-3-like 1; CRP, C-reactive protein; IL-6, interleukin 6; IL-18BP, interleukin 18 binding protein; sICAM-1, soluble intercellular adhesion molecule-1; sTNFR2, soluble tumor necrosis factor receptor 2; LR, likelihood ratio; SD, standard deviation; SE, standard error.

Supplementary Table S4: Linear mixed effects modeling of longitudinal changes in inflammatory proteins across pregnancy by malaria status

	CHI3L1		CRP		IL-18BP		IL-6		sICAM-1		sTNFR2	
**Fixed Effects[a]**	**Estimate (SE)**	**T-Value**	**Estimate (SE)**	**T-Value**	**Estimate (SE)**	**T-Value**	**Estimate (SE)**	**T-Value**	**Estimate (SE)**	**T-Value**	**Estimate (SE)**	**T-Value**
(Intercept)	4.24 (0.47)	8.94	0.67 (0.79)	0.85	2.44 (0.23)	10.72	0.78 (0.29)	2.75	5.66 (0.17)	33.95	2.12 (0.26)	8.17
Malaria	-0.07 (0.10)	-0.70	-0.13 (0.16)	-0.82	0.16 (0.05)	3.39	0.04 (0.06)	0.74	0.07 (0.04)	2.05	0.12 (0.054)	2.28
Gestational Age (Shifted)	-0.009 (0.005)	-1.79	0.01 (0.01)	1.51	0.005 (0.002)	2.23	-0.007 (0.006)	-1.29	-0.006 (0.002)	-3.38	-0.003 (0.003)	-0.89
Maternal Age	0.02 (0.01)	1.94	0.02 (0.02)	0.90	0.001 (0.006)	0.19	-0.007 (0.008)	-0.87	0.002 (0.005)	0.51	-0.009 (0.007)	-1.31
BMI at Enrollment	-0.04 (0.02)	-2.45	0.009 (0.03)	0.31	-0.005 (0.008)	-0.57	0.004 (0.01)	0.38	-0.01 (0.006)	-2.32	-0.012 (0.009)	-1.29
Gravidity	0.008 (0.03)	0.27	-0.11 (0.05)	-2.11	0.02 (0.016)	1.45	-0.01 (0.018)	-0.54	-0.006 (0.01)	-0.50	0.031 (0.017)	1.81
Gestational age; treatment arm interaction	0.003 (0.005)	0.55	-0.02 (0.009)	-1.66	0.001 (0.003)	0.45	-0.002 (0.004)	-0.46	0.001 (0.002)	0.74	0.004 (0.003)	1.16
**Random Effects**	**Variance (SD)**	**----**	**Variance (SD)**	**----**	**Variance (SD)**	**----**	**Variance (SD)**	**----**	**Variance (SD)**	**----**	**Variance (SD)**	**----**
Participant (Intercept)	1.26 (1.12)	----	2.00 (1.41)	----	0.14 (0.37)	----	0.023 (0.15)	----	0.06 (0.25)	----	0.16 (0.40)	----
Gestational Age (Shifted)	0.0007 (0.03)	----	0.0005 (0.024)	----	0.0002 (0.013)	----	0.0004 (0.02)	----	0.00001 (0.004)	----	0.0001 (0.01)	----
Residual	0.33 (0.58)	----	1.64 (1.28)	----	0.07 (0.26)	----	0.73 (0.85)	----	0.05 (0.22)	----	0.19 (0.44)	----
Observations (n)	1029	----	1029	----	916	----	1029	----	916	----	1029	----
Participants (n)	293	----	293	----	264	----	293	----	264	----	293	----
LR test against null model	$X^2(1) = 0.50$, P = 0.48		$X^2(1) = 0.67$, p = 0.41		$X^2(1) = 11.45$, **p < 0.001**		$X^2(1) = 0.53$, p = 0.47		$X^2(1) = 4.23$, **p = 0.04**		$X^2(1) = 5.24$, **p = 0.02**	

Linear mixed effects modeling to evaluate changes in inflammatory proteins across pregnancy in malaria infected and uninfected pregnancies. [a]Models adjusted for gestational age at blood sample collection, maternal age, BMI at enrollment, gravidity, and the interaction between treatment arm and gestational age. Abbreviations: BMI, body mass index; CHI3L1, chitinase-3-like 1; CRP, C-reactive protein; IL-6, interleukin 6; IL-18BP, interleukin 18 binding protein; sICAM-1, soluble intercellular adhesion molecule-1; sTNFR2, soluble tumor necrosis factor receptor 2; LR, likelihood ratio; SD, standard deviation; SE, standard error.

Supplementary Table S5: Linear mixed effects modeling of longitudinal changes in inflammatory proteins across term and preterm pregnancies

	CHI3L1		CRP		IL-18BP		IL-6[b]		sICAM-1		sTNFR2	
**Fixed Effects[a]**	**Estimate (SE)**	**T-Value**	**Estimate (SE)**	**T-Value**	**Estimate (SE)**	**T-Value**	**Estimate (SE)**	**T-Value**	**Estimate (SE)**	**T-Value**	**Estimate (SE)**	**T-Value**
(Intercept)	4.35 (0.45)	9.56	1.15 (0.75)	1.52	2.67 (0.22)	12.06	0.84 (0.28)	3.04	5.77 (0.16)	36.28	2.25 (0.25)	8.94
Preterm	0.16 (0.13)	1.28	-0.02 (0.21)	-0.12	0.06 (0.06)	0.96	-0.38 (0.18)	-2.08	0.08 (0.04)	1.72	0.15 (0.07)	2.20
Gestational Age (Shifted)	-0.011 (0.006)	-1.86	0.004 (0.01)	0.36	0.002 (0.003)	0.75	-0.002 (0.007)	-0.32	-0.009 (0.002)	-4.35	-0.002 (0.004)	-0.55
Treatment group	-0.12 (0.17)	-0.70	-0.35 (0.27)	-1.29	-0.08 (0.07)	-1.28	0.25 (0.13)	1.85	-0.108 (0.05)	-2.18	0.05 (0.09)	0.52
Maternal Age	0.02 (0.01)	1.60	0.009 (0.02)	0.44	0.0004 (0.006)	0.07	-0.005 (0.007)	-0.63	0.004 (0.004)	0.97	-0.007 (0.007)	-1.15
BMI at Enrollment	-0.04 (0.016)	-2.44	0.006 (0.03)	0.21	-0.006 (0.008)	-0.83	-0.002 (0.01)	-0.25	-0.016 (0.006)	-2.83	-0.02 (0.009)	-1.79
Gravidity	0.02 (0.03)	0.61	-0.09 (0.05)	-1.99	0.013 (0.014)	0.88	-0.019 (0.017)	-1.14	0.014 (0.010)	-1.40	0.015 (0.015)	0.99
Gestational age; treatment arm interaction	0.006 (0.008)	0.76	0.001 (0.02)	0.06	0.006 (0.004)	1.61	-0.02 (0.01)	-1.98	0.007 (0.003)	2.58	0.001 (0.006)	3.44
**Random Effects**	**Variance (SD)**	**----**	**Variance (SD)**	**----**	**Variance (SD)**	**----**	**Variance (SD)**	**----**	**Variance (SD)**	**----**	**Variance (SD)**	**----**
Participant (Intercept)	1.29 (1.14)	----	1.94 (1.39)	----	0.15 (0.38)	----	0.01 (0.10)	----	0.06 (0.24)	----	0.18 (0.42)	----
Gestational Age (Shifted)	0.0008 (0.03)	----	0.0006 (0.02)	----	0.0002 (0.01)	----	0.0002 (0.016)	----	0.000004 (0.002)	----	0.0002 (0.012)	----
Residual	0.34 (0.58)	----	1.59 (1.26)	----	0.07 (0.26)	----	0.72 (0.85)	----	0.05 (0.22)	----	0.19 (0.43)	----
Observations (n)	1102	----	1102	----	985	----	1102	----	985	----	1102	----
Participants (n)	319	----	319	----	289	----	319	----	289	----	319	----
LR test against null model	$X^2(1) = 1.65$, p = 0.20		$X^2(1) = 0.01$, p = 0.91		$X^2(1) = 0.94$, p = 0.33		$X^2(1) = 5.79$, **p = 0.02**		$X^2(1) = 3.00$, p = 0.08		$X^2(1) = 4.90$, **p = 0.03**	

Linear mixed effects modeling (LME) to evaluate changes in inflammatory proteins across pregnancy in term and preterm pregnancies. [a]Models adjusted for gestational age at blood sample collection, treatment group, maternal age, BMI, gravidity, and the interaction between treatment arm and gestational age. [b]The LME model for IL-6 included an interaction term between gestational age and preterm birth outcome (Estimate (SE): 0.036 (0.015); T-value: 2.41). Abbreviations: BMI, body mass index; CHI3L1, chitinase-3-like 1; CRP, C-reactive protein; IL-6, interleukin 6; IL-18BP, interleukin 18 binding protein; sICAM-1, soluble intercellular adhesion molecule-1; sTNFR2, soluble tumor necrosis factor receptor 2; LR, likelihood ratio; SD, standard deviation; SE, standard error.

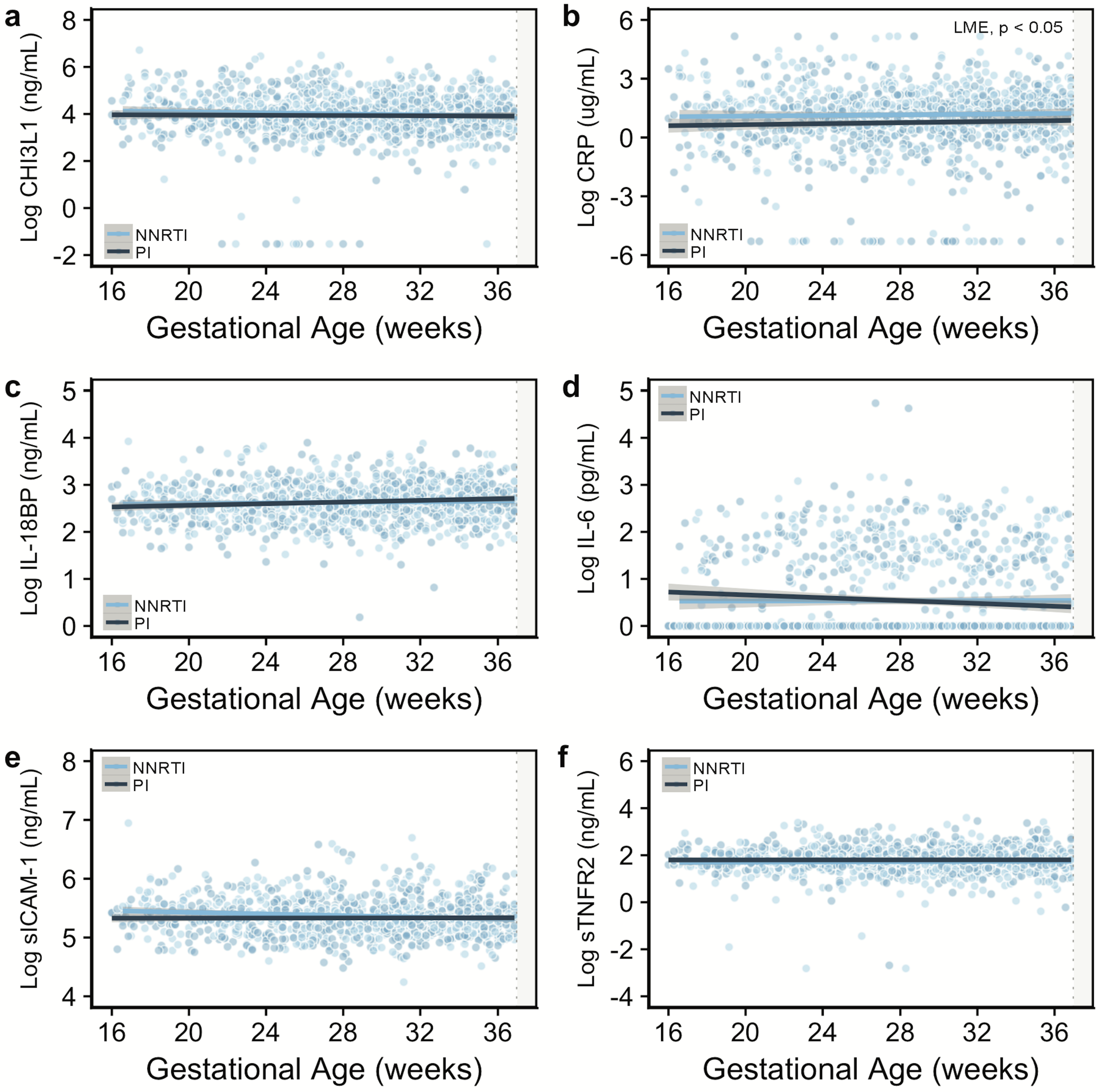

Supplementary Figure S1: Longitudinal changes in inflammatory markers across pregnancy by parent trial treatment arm. Logged (natural log) plasma concentrations of (a) CHI3L1 (ng/mL), (b) CRP (μg/mL), (c) IL-18BP (ng/mL), (d) IL-6 (pg/mL), (e) sICAM-1 (ng/mL), and (f) sTNFR2 (ng/mL) by gestational age of sample collection. Abbreviations: CHI3L1, chitinase-3-like 1; CRP, C-reactive protein; IL-6, interleukin-6; IL-18BP, interleukin 18 binding protein; sICAM-1, soluble intercellular adhesion molecule-1; sTNFR2, soluble tumor necrosis factor receptor-2.
